# A Semiquantitative Non-invasive Measurement of PcomA Patency in C57BL/6 Mice Explains Variance in Ischemic Brain Damage in Filament MCAo

**DOI:** 10.3389/fnins.2020.576741

**Published:** 2020-09-23

**Authors:** Samuel Knauss, Carolin Albrecht, Ulrich Dirnagl, Susanne Mueller, Christoph Harms, Christian Johannes Hoffmann, Stefan Paul Koch, Matthias Endres, Philipp Boehm-Sturm

**Affiliations:** ^1^Department of Neurology with Experimental Neurology, Charité-Universitätsmedizin Berlin, Corporate Member of Freie Universität Berlin, Humboldt-Universität zu Berlin, Berlin Institute of Health, Berlin, Germany; ^2^German Center for Cardiovascular Research (DZHK), Partner Site Berlin, Berlin, Germany; ^3^Center for Stroke Research Berlin, Charité-Universitätsmedizin Berlin, Berlin, Germany; ^4^NeuroCure Clinical Research Center, Charité - Universitätsmedizin Berlin, Berlin, Germany; ^5^German Center for Neurodegenerative Diseases (DZNE), Bonn, Germany; ^6^Quality, Ethics, Open Science, Translation, Center for Transforming Biomedical Research, Berlin Institute of Health, Berlin, Germany; ^7^NeuroCure Cluster of Excellence and Charité Core Facility 7T Experimental MRIs, Charité – Universitätsmedizin Berlin, Berlin, Germany

**Keywords:** stroke, mouse, angiography, MRI, posterior communicating artery, lesion size

## Abstract

Numerous studies on experimental ischemic stroke use the filament middle cerebral artery occlusion (fMCAo) model in C57BL/6 mice, but lesion sizes in this strain are highly variable. A known contributor is variation in the posterior communicating artery (PcomA) patency. We therefore aimed to provide a semiquantitative non-invasive *in vivo* method to routinely assess PcomA patency. We included 43 male C57BL/6 mice from four independent studies using a transient 45 min fMCAo model. Edema-corrected lesion sizes were measured by magnetic resonance (MR) imaging 24 h after reperfusion. Time-of-flight MR angiography was performed 7 days before and 24 h after fMCAo. Scores of PcomA size measured 24 h after, but not scores measured 7 days before fMCAo were negatively correlated with lesion size. Variability in PcomA patency explained 30% of the variance in our cohort (*p* < 0.0001, coefficient of determination *r*^2^ = 0.3). In a simulation using parameters typical for experimental stroke research, the power to detect a true effect of *d* = 1 between two groups increased by 15% when an according covariate was included in the statistical model. We have demonstrated that *in vivo* measurement of PcomA size is feasible and can lead to increased accuracy in assessing the effect of treatments.

## Introduction

Animal models of focal cerebral ischemia are a cornerstone of stroke research, as indicated by over 1.000 experimental treatments for acute stroke that have been evaluated in these models (O’Collins et al., 2006). A prerequisite for a successful translation of preclinical findings into clinical trial is selection of the correct target in preclinical experiments (Dirnagl and Macleod, 2009; Sena et al., 2010). Possibly the greatest risk to reproducibility of results and correct target selection comes from the omnipresence of small sample sizes and correspondingly low statistical power (Button et al., 2013). Low statistical power can lead to overestimation of effect sizes and low positive predictive value of positive findings but also increases the risk of overlooking the true effects of therapeutics at an early stage (Button et al., 2013; Munafò et al., 2014). Here, we propose a method to increase power and reduce noise in one of the most widely used stroke models and one of the most common animal strains.

The intraluminal filament method of middle cerebral artery occlusion (fMCAo) is among the most commonly used methods to induce focal ischemic stroke in animal studies (Morris et al., 2016). In order to reduce the imbalance of covariates in preclinical stroke models, standard operating procedures and refinements to the methods have been formulated to control known determinants of outcome variables (Connolly et al., 1996; Dirnagl, 2012; Morris et al., 2016). Often, the variance in lesion size, which is one of the main outcome parameters, is as high as 40% of the mean, rendering the generation of robust and reproducible data with prevalently used small sample sizes difficult (Dirnagl, 2006). The extent of this variability greatly depends on the mouse strain used in the experiment (Yang et al., 1997; Kitagawa et al., 1998). One of the most commonly used mouse strains, but at the same time one of the strains with the largest variability in lesion size, is C57BL/6, due to its widespread use for generating genetically modified mice (Kitagawa et al., 1998; Maeda et al., 1998; McColl et al., 2004). One of the factors determining the variability in lesion size is the patency of the circle of Willis in this strain (Kitagawa et al., 1998; Maeda et al., 1998; McColl et al., 2004). Blood supply to the rodent brain is provided through four main arteries: two vertebral arteries posteriorly and two carotid arteries anteriorly. At the base of the brain, these arteries are connected by anastomoses, forming the circle of Willis, with two posterior communicating arteries and one anterior communicating artery. In 1998, Kitagawa showed that the patency of the posterior communicating artery (PcomA) is a crucial determinant of the lesion size in the fMCAo model (Kitagawa et al., 1998). Of note, nomenclature for the communicating arteries in mice is inconsistent in the literature. In this paper, we adhered to the nomenclature used in the work published by Kitagawa et al. (1998) describing the connecting artery between the superior cerebellar artery (SCA) and the posterior cerebral artery (PCA) as PcomA, which corresponds to the P1 segment of the human PCA (Xiong et al., 2017).

In this study, we sought to establish an *in vivo* imaging approach to assess the patency and size of the PcomA as a determinant of lesion size, to be used in adjusting for pre-existing differences in this important covariate. Including this variable in the statistical model to test for treatment effects in preclinical stroke models might increase power and precision to detect true effects and arrive at more precise estimates of effect sizes.

## Materials and Methods

### Animals and Experimental Groups

All experimental procedures were approved by the “Landesamt für Gesundheit und Soziales Berlin” under registration numbers G0005/16, G0065/17 and G0261/17 and were in accordance with national and institutional guidelines for the care and use of laboratory animals and with directive 2010/63/EU of the European Parliament and of the Council of 22 September 2010. The reporting conforms to the ARRIVE guidelines (Kilkenny et al., 2010). A total of 33 C57BL/6J (group 1–3) and 10 C57BL/6N mice (group 4) (10 weeks old, mean weight 25 ± 2 g) were used from control groups of four studies testing different neuroprotective treatments. C57BL/6J and C57BL/6N mice were purchased from Charles River Germany, and the animals were housed in groups of 3–5 per cage with a standard light-dark cycle (12 h:12 h) and *ad libitum* access to food and water. Animals were rested for 7 days upon arrival to the laboratory. Investigating the association between PcomA patency and lesion size was pre-specified as an independent study goal in the study protocols. The animals included in the analysis for the present study were either naïve or treated with a vehicle depending on the respective group (non-reactive mGo53 Antibodies (Wardemann et al., 2003) for group 1 and 3, phosphate buffered saline for group 2 and no treatment for group 4. Group 1 (*n* = 10) was used to establish the method for MRA acquisition and semiquantitative measurement of PcomA size 24 h after fMCAo described below. Five animals in group 1 were used to study PcomA size *ex vivo* by Evans Blue perfusion after MRA acquisition. Group 2 and group 3 (group 2: *n* = 13; group 3: *n* = 10) underwent the same protocol of 45 min fMCAo followed by magnetic resonance imaging (MRI) and magnetic resonance angiography (MRA) 24 h after fMCAo. The sample size for group 2 was calculated assuming an effect size of *r*^2^ = 0.6 at an α = 0.05 and a power level of 0.8 based on results from group 1.

**FIGURE 1:**
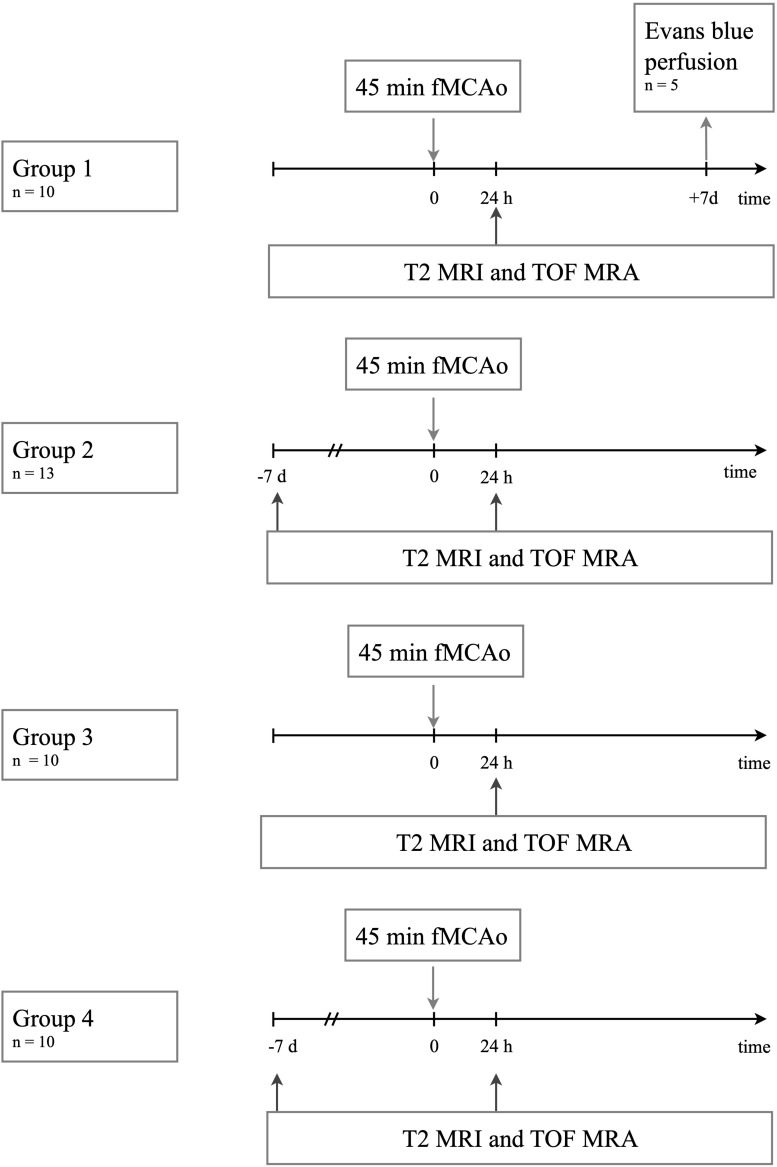
Experiments performed per experimental group.

To test the robustness of the method, we included an additional group from another experimental hardware setup (group 4: *n* = 10). The protocols for 45 min fMCAo were the same in group 4 as in the other groups. The fMCAo surgery and MRI / MRA acquisition were performed by two independent researchers. Groups 2 and 4 underwent additional baseline MRA scans 7 days before fMCAo. Figure 1 gives an overview of the experiments included in this study.

### Randomization, Blinding, and Exclusion Criteria

For randomization, animals were numbered, and operation order was randomly assigned. For all protocols and outcome assessments, the experimenters were blinded. All outcome assessments were performed by observers blinded to previous treatments and outcomes. All animals included in the study (*n* = 43) were included in the analyses.

### Model of Middle Cerebral Artery Occlusion in Mice

All experimental procedures were conducted by a trained experimenter following published standard operating procedures (Dirnagl, 2012). A transient fMCAo model was generated as described previously (Engel et al., 2011). Mice were anesthetized using 2.5% (vol/vol) isoflurane for induction and 1.5% (vol/vol) isoflurane for maintenance, mixed in both cases with 30% O_2_ and ∼68% N_2_O. After permanent occlusion of the ipsilateral external carotid and the common carotid artery, cerebral ischemia was induced by introducing a 7-0 silicon-rubber-coated fMCAo suture with a coating length of 9–10 mm (monofilament 7019910PK5Re, Doccol Corp., Sharon MA, United States) into the left internal carotid artery and advancing it up to the anterior cerebral artery, thereby occluding the middle cerebral artery. The filament was withdrawn after an occlusion time of 45 min. Rectal temperature was maintained at 37.5 ± 0.5°C throughout the experiment using a feedback-controlled heating pad with a rectal probe (Fine Science Tools GmbH, Heidelberg, Germany). During fMCAo and for at least 2 h after reperfusion, animals were allowed to recover in a heated cage (Peco Services, Cumbria, United Kingdom). Afterward, they were transferred to the home cage. After fMCAo, animals had access to soft food and were treated with subcutaneous saline injections to ensure adequate hydration after surgery.

### Evans Blue Perfusion

For Evans Blue perfusion and vessel fixation, mice were deeply anaesthetized with ketamine and xylazine at the conclusion of the experiment and perfused through the heart with physiological saline followed by 4% paraformaldehyde, Termofisher, W32465, 3% Gelatin (Sigma-Aldrich, G1890), 1% low melting agarose (Sigma Aldrich A4018) and 0.1% Evans Blue (Sigma Aldrich E2129) as described previously (Foddis et al., 2019).

### Magnetic Resonance Imaging

Magnetic resonance imaging (MRI) was conducted on a 7-Tesla rodent scanner running Paravision 5.1 software (Pharmascan 70/16, Bruker, Ettlingen, Germany) with a 16 cm horizontal bore magnet and a 9 cm (inner diameter) shielded gradient with a maximum gradient strength of 300 mT/m. Mice were anesthetized under 1.5% isoflurane in a mixture of 30% O_2_/68.5% N_2_O and scanned under constant respiration monitoring (Small Animal Monitoring & Gating System, SA Instruments, Stony Brook, New York, United States). Animals were placed prone and head fixed on a heated circulating water blanket to ensure a constant body temperature of 37 ± 0.5°C.

For groups 1–3, a 72 mm volume resonator (RAPID Biomedical, Rimpar, Germany) was used for transmission, and an actively decoupled mouse head surface coil was used for signal reception. After coil tuning and matching, scout images were acquired. Similar geometry of all scans across animals was assured by using the anterior commissure as a landmark in rostral-caudal direction and the trachea as a landmark in the other directions. T2-weighted images were acquired using a 2D turbo spin-echo sequence (2D RARE) with repetition time (TR) = 4200 ms, echo time (TE) = 36 ms, echo spacing (ΔTE) = 12 ms, RARE factor = 8, 4 averages, 32 contiguous axial slices with a slice thickness of 0.5 mm, field of view (FOV) = 25.6 × 25.6 mm^2^, matrix size MTX = 256 × 256, bandwidth BW = 46875 Hz, and total acquisition time TA = 6 min 43 s). Angiography with matching geometry but higher resolution was performed with a non-flow compensated 3D-FLASH time-of-flight (TOF) sequence with TR/TE = 15 ms/2.5 ms, 1 ms sinc-shaped excitation pulse with 22 kHz bandwidth and flip angle FA = 20°, FOV = 25.6 × 25.6 × 16.0 mm^3^, MTX = 256 × 223 × 139 zero filled to 256 × 256 × 160, BW = 98684 Hz, TA = 7 min 50 s.

For group 4, a transmit/receive quadrature volume resonator with an inner diameter of 20 mm (RAPID) was used. T2-weighted images were acquired with the same sequence as groups 1–3. Angiography with FOV centered to the FOV of the T2-weighted sequence was acquired with a 3D-FLASH TOF using identical settings as for groups 1–3 but TR/TE = 14 ms/2.3 ms, FOV = 19.2 × 19.2 × 16.0 mm^3^, MTX = 192 × 167 × 139 zero filled to 192 × 192 × 160, TA = 5 min 29 s.

### Atlas Registration

T2-weighted images were registered to the Allen Brain Atlas^1^ as described previously (Koch et al., 2017) using ANTX^2^, a custom MATLAB toolbox comprising a segmentation in SPMMouse^3^ followed by non-linear warping of tissue probability maps in ELASTIX^4^. The calculated image transformation could then be applied to other images aligned with the T2-weighted scan, including TOF angiograms and lesion masks.

### Corrected Lesion Volume

Lesion volume corrected for brain swelling was calculated as described previously (Koch et al., 2017). Briefly, ischemic tissue appearing hyperintense on T2-weighted images 24 h after stroke was manually delineated by an experienced researcher, carefully omitting ventricles, using ANALYZE 5.0 software (AnalyzeDirect, Overland Park, KS, United States), and masks of the same dimensionality as the T2-weighted image were exported in NIFTI format. Using nearest neighbor interpolation, lesion masks were registered to the Allen Brain Atlas. Edema-corrected lesion volume in percent was defined as the volume of the lesion divided by volume of the hemisphere measured in atlas space.

### Semiquantitative Assessment of PcomA Patency

ImageJ distribution 1.51m9 (Schneider et al., 2012) was used for post-processing atlas-registered angiograms to obtain scores for PcomA patency and size. Atlas registration allowed us to determine a set of 10 slices that contained the posterior part of the circle of Willis in all animals. To render pixel intensity comparable between individual datasets, we then calculated the signal-to-noise ratio (SNR) of each dataset adjusted as described previously (Gudbjartsson and Patz, 1995; Boehm-Sturm et al., 2011) and set a threshold to 1/10 of the maximum intensity of the dataset, setting all other values to NaN. In the next step, the area between the superior cerebellar artery (SCA) and posterior cerebral artery (PCA) was manually delineated on all selected slices. The maximum SNR in this area depends on background intensity and on the flow signal present, with higher intensities for higher flow. The only larger vessel in this anatomical area is the PcomA (Xiong et al., 2017). We therefore used the maximum SNR measured over the stack of slices as a correlate and semiquantitative score of the flow and thus the patency of the PcomA. Only the side ipsilateral to the ischemic lesion was assessed. ImageJ code is available on www.figshare.com at https://www.doi.org/10.6084/m9.figshare.8950328.

### Validation by Manual *in vivo* and *ex vivo* Assessments of PcomA

The semiquantitative assessment of PcomA patency was correlated to an *in vivo* score of PcomA size in the described target region assessed and rated from 0 (meaning no vessel) to 3 (large vessel) using the same postprocessing as described above.

To quantitatively validate *in vivo* assessment of PcomA size, a subset of animals was perfused with Evans Blue and *ex vivo* photographs of excised brains were taken. PcomA size was measured in percent of the basilar artery *in vivo* on maximum intensity projections of raw TOF images and *ex vivo* on Evans Blue labeled vasculature.

### Simulation

A statistical program was developed in STATA 13.0 (StataCorp, College Station, United States) to simulate a hypothetical two-arm trial testing the effect of a hypothetical treatment. The simulation code was based on work by Egbewale et al. (2014) describing the method to simulate data for clinical trials. Based on assumptions common in preclinical stroke research (Howells et al., 2012), we set the standardized effect size at *d* = 1.0. Setting the α error probability at 0.05 and the power at 0.8, we set the sample size at *n* = 14 per group. A normally distributed covariate with varying degrees of correlation with the outcome variable was introduced. Five different experimental conditions with correlation coefficients between the covariate and the outcome variable of *r*^2^ = 0, 0.01, 0.04, 0.09, 0.16, 0.25, 0.29, 0.36, 0.49, and 0.64 were simulated. Outcome analysis was performed by either ANOVA or ANCOVA adjusting for the covariate in each individual experiment. Each hypothetical scenario and outcome analysis combination was repeated 1000 times to obtain reliable estimates of actual power. STATA code is available on www.figshare.com at https://www.doi.org/10.6084/m9.figshare.9752834.

### Statistical Evaluation

Data are represented as the Pearson correlation coefficient r or as the coefficient of determination r^2^ and calculated probability value (*p*-value) using the general linear model. For testing of manual ratings measured on an ordinal scale, data are represented as Spearman rank-order correlation coefficient r_S_.

### Data Availability

Source code for the simulation in STATA and the code for the macro used to postprocess images for semiquantitative measurement in ImageJ are available in the Appendix. All relevant original and Allen Brain Atlas-registered images are publicly available at http://doi.org/10.6084/m9.figshare.9752864.

## Results

### TOF MRA Can Image PcomA Size After fMCAo

**FIGURE 2:**
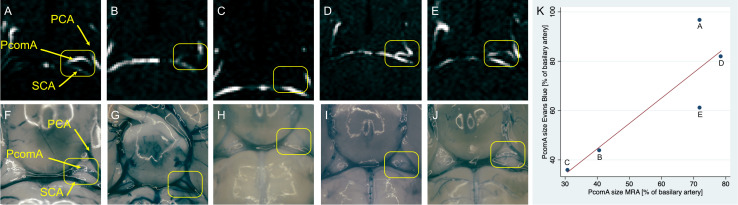
TOF MRA images and corresponding images of vasculature after Evans Blue perfusion. TOF-MR angiography 24 h after fMCAo readily images the circle of Willis and PcomA **(A–E)**. Evans Blue perfusion 7 days after fMCAo clearly depicts the corresponding vasculature **(F–J)**. Yellow boxes highlight the PcomA ipsilateral to the ischemic lesion. Individual data points of images **(A–J)**, respectively, comparing readings of PcomA diameter as percentage of basilar artery diameter from MRA and from images after Evans Blue perfusion **(K)**. PCA – posterior cerebral artery, PcomA – posterior communicating artery, SCA – superior cerebellar artery.

PcomA size was assessed *ex vivo* 7 days after fMCAo in five animals of group 1 (Figure 2). *Ex vivo* PcomA vessel size was measured after Evans Blue perfusion in relation to the size of the basilar artery and compared to corresponding measurements obtained *in vivo* 24 h after fMCAo by MR angiography. PcomA size measured by MRA was highly correlated (*n* = 5, *r*^2^ = 0.81) with *ex vivo* PcomA size measurements, yet statistical significance was not reached (*p* = 0.057).

### Semiquantitative Measurement of PcomA Patency by MRA Is Correlated With Manual PcomA Size Ratings

**FIGURE 3:**
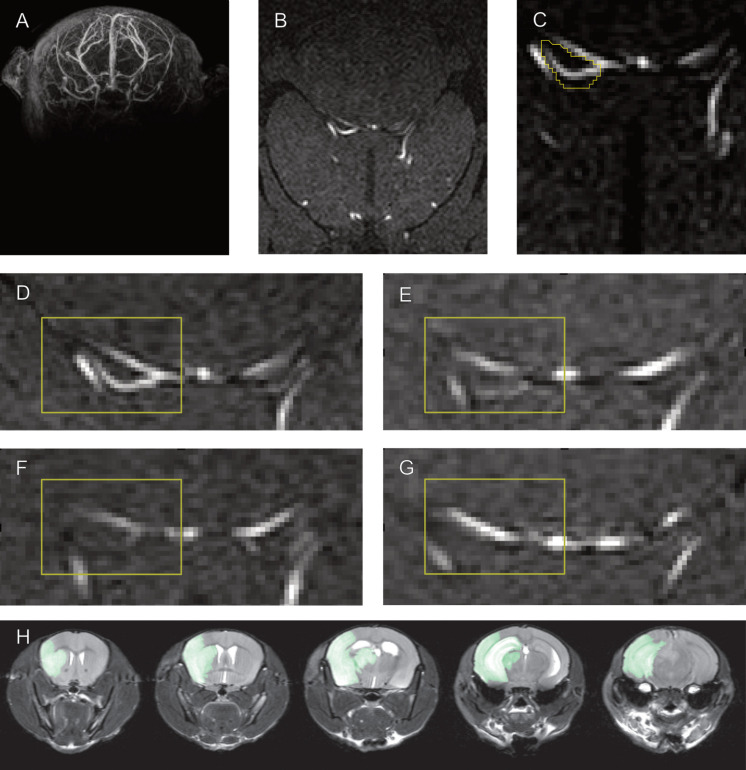
Semiquantitative measurement of PcomA size and manual PcomA assessment. Whole brain TOF MRA recordings **(A)** were normalized to the Allen Brain atlas **(B)** to define a set of slices that contain the location of the PcomA in all animals. Semiquantitative measurements of PcomA patency were taken by assessing the maximum SNR in a manually delineated region of interest between the SCA and the PCA **(C)** in these slices. Examples of Allen atlas-normalized maximum intensity projections with large **(D)**, medium **(E)**, small **(F)** and absent **(G)** PcomA used for the training of raters to manually rate PcomA size. Examples of T2 MRI images used to obtain a mask of the ischemic lesion **(H)**.

To validate the semiquantitative measurement of PcomA patency with an independent data analysis method, we compared semiquantitative measurements to independently conducted manual ratings (Figure 3). All imaging studies followed the same procedure of atlas registration before being further processed for unbiased semiquantitative measurement. The same atlas-registered datasets were used for evaluation by three trained raters blinded to the total lesion volume. Raters were trained with a standard set from group 1. There was a good correlation of manually rated PcomA size and semiquantitative measurement of PcomA patency (Spearman rank-order correlation coefficient r_S_ = 0.7, *p* < 0.0001). Ratings for the size of the PcomA on each side varied between independent raters, but interrater reliability was high (interclass correlation = 0.9, *p* < 0.001).

### Semiquantitative Measurement of PcomA Patency Partially Explains Variability in Lesion Size

**FIGURE 4:**
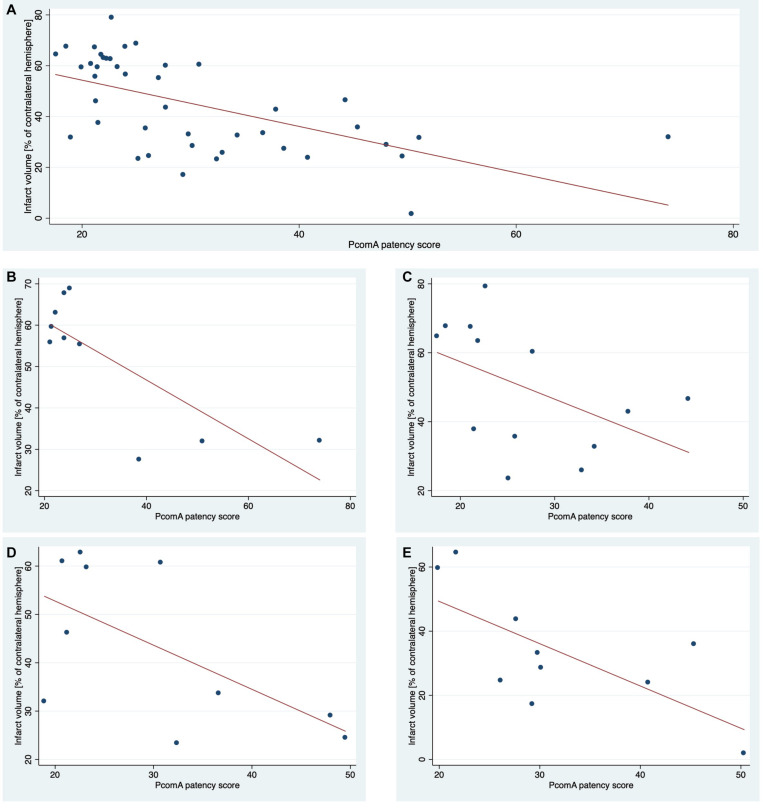
Correlation of PcomA score and lesion size 24 h after fMCAo. Lesion sizes of each animal included in this study and corresponding semiquantitative scores of PcomA patency **(A)**. A method to obtain PcomA scores was developed using group 1 **(B)**. The results were replicated in three additional independent groups 2–4 **(C–E)**.

**FIGURE 5:**
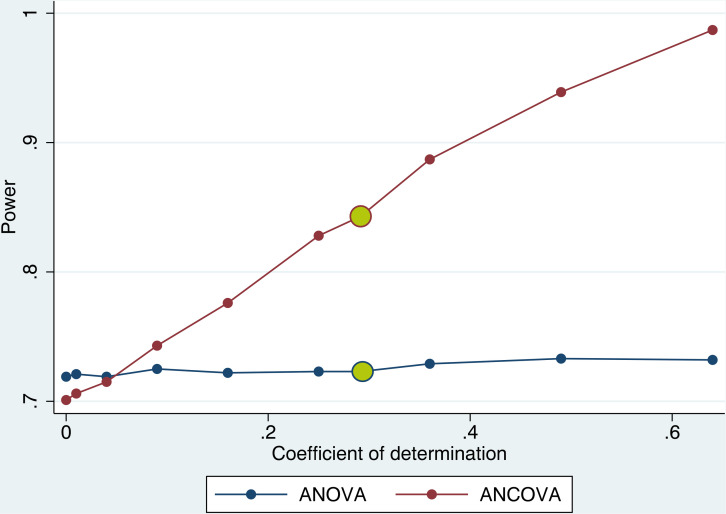
Power to detect an effect of *d* = 1 with and without adjusting for a covariate. Adjusting for a single covariate in the outcome analysis (ANCOVA) increased the power to detect a true effect by up to 25% compared to no adjustment (ANOVA) depending on the magnitude of the correlation coefficient of covariate and outcome variable. Effect size *d* = 1, *n* = 14 per group, 1000 repetitions per condition. Coefficient of determination found in this study for the association of PcomA score and lesion size marked in yellow (*r*^2^ = 0.34).

Unbiased semiquantitative measurement of PcomA patency from TOF angiograms recorded at 24 h after fMCAo significantly negatively correlated with lesion size determined by T2 weighted MRI at 24 h (Figure 4) in 4 independent datasets. In the first training set, 62% of intersubject variability of total lesion size was explained by PcomA patency (*r*^2^ = 0.62) alone. Applying this algorithm to three independent datasets, 16–49% (*r*^2^ = 0.16–0.49) (Table 1) of variability in lesion size in these individual groups was explained by differences in semiquantitative PcomA patency measurements. Pooling data from all groups resulted in a highly significant correlation explaining 34% of the variance in the dataset, *r*^2^ = 0.34, *p* < 0.0001 (Figure 5).

**TABLE 1:** Correlation of semiquantitative scores of PcomA patency and lesion size 24 h after fMCAo.

Group	*n*	Correlation coefficient (*R*)	Coefficient of determination (*R*^2^)	*p*
Group 1	10	–0.76	0.62	<0.01
Group 2	13	–0.4	0.16	0.09
Group 3	10	–0.61	0.38	0.05
Group 4	10	–0.7	0.49	0.023
All Groups	43	–0.58	0.34	<0.0001

### Semiquantitative Measurement of PcomA Patency Prior to fMCAo Cannot Reliably Predict Infarct Size

There was a moderate correlation between mean manual PcomA size ratings before fMCAo and unbiased semiquantitative measurement of PcomA patency 24 h after fMCAo (*r* = 0.63, *r*^2^ = 0.40, *p* = 0.001). While there was still a trend for a moderate correlation between mean manual PcomA size ratings before fMCAo and MRI lesion size 24 h after fMCAo (Spearman rank-order correlation coefficient *r* = −0.40, *p* = 0.051), semiquantitative PcomA measurement before fMCAo did not reliably predict lesion size across 2 datasets (group 2 and group 4; *r* = −0.0186, *r*^2^ = 0.0003, *p* = 0.933). For group 1 and group 3, no baseline MRA before fMCAo was recorded.

### Including PcomA Size as a Covariate Increases Power in a Simulation Preclinical Stroke Study

We conducted a simulation experiment to test whether the power of the final outcome analysis increases by including a covariate with the magnitude of correlation found for the association between PcomA patency and lesion size in this study. In this simulation, the power to detect an effect with a standardized effect size of *d* = 1 increased by 12% when adjusting for a covariate with a correlation coefficient of *r*^2^ = 0.25 and further increased for stronger associations between the covariate and the outcome variable (Figure 5).

### Distribution of PcomA Phenotypes in C57BL/6 Mice and Correlation With Lesion Size

The vascular anatomy was examined using TOF MRA images recorded 24 h after fMCAo and separate manual ratings of whether the PcomA was patent on each side. In our cohort, we found a complete circle of Willis in 21% (9/43), a patent PcomA on one side in 53% (23/43) and a bilateral lack of patent PcomAs in 26% (11/43) of animals. Ischemic lesion size was significantly correlated with PcomA phenotype, with smaller lesion sizes in animals with a complete circle of Willis (*r*^2^ = 0.32, *p* = 0.0001).

## Discussion

We developed an easy-to-implement semiquantitative method to assess PcomA patency *in vivo* using readily available standard mouse MRI sequences. We demonstrated that the obtained dimensionless score negatively correlates with the lesion size after fMCAo in C57BL/6 mice. In a simulation experiment representative of common study designs in preclinical stroke research, we showed that by including this score as a covariate in the model, the power to detect a true effect increased by almost 20%.

The fMCAo model is widely used to model human focal cerebral ischemia (Kleinschnitz et al., 2015). One of its inherent disadvantages is the high intersubject variability of common outcome parameters. Several variables have been shown to impact lesion volume, one of the most important outcome parameters (Morris et al., 2016); including different parameters of the model itself, such as the occlusion and recovery time (Harada et al., 2005; Liu et al., 2009), the filament and anesthetic used (Michenfelder et al., 1976; Tsuchiya et al., 2003), differences in the surgical technique (Chen et al., 2008) as well as parameters of the animals, such as the species (Carmichael, 2005), strain (Yang et al., 1997; Kitagawa et al., 1998), substrain (Zhao et al., 2019), sex (Alkayed et al., 1998; Zhao et al., 2019), age (Sutherland et al., 1996), and interindividual variation in cerebrovascular anatomy (Barone et al., 1993; Kitagawa et al., 1998; McColl et al., 2004). Species and strain differences in the cerebrovascular architecture and in particular differences in the patency of macroanastomoses formed in the circle of Willis have already been studied previously (Barone et al., 1993; Kitagawa et al., 1998; McColl et al., 2004). In a study in 1998, Kitagawa and colleagues demonstrated species and strain differences of the circle of Willis in mice (Kitagawa et al., 1998; Ankolekar et al., 2012). In the same study, they were able to establish patency of the PcomA as a determinant of parenchymal ATP drop in a model of bilateral carotid artery occlusion and mortality and lesion size in fMCAo. To predict the vascular anatomy, they proposed a method using laser Doppler flow measurement of cerebral microvascular perfusion and a one-minute bilateral common carotid artery (CCA) occlusion. However, this method could neither determine whether the PcomA was patent on one or both sides nor detect the side of a unilaterally patent PcomA. The authors concluded that PcomA patency should be assessed for every animal in models of cerebral ischemia, but that their own method could be used only to predict forebrain ischemia after bilateral CCA occlusion and not for unilateral MCAo. Additional evidence of the impact of PcomA patency and the variability of PcomA patency in C57BL/6J mice comes from McColl and colleagues: in a small sample of 10 C57BL/6J mice, they found a complete circle of Willis in only 1 animal. In 6 animals, the PcomA was patent only unilaterally, and in another 3 animals, no patent PcomA was found (McColl et al., 2004). In our own cohort of 43 animals, we found a slightly different distribution: we confirmed that a plurality of animals had a single patent PcomA, while almost equal numbers of animals had bilaterally patent PcomAs and complete absence of the posterior part of the circle of Willis. The notion of PcomA patency being one of the main determinants of mortality and lesion size in the fMCAo model is corroborated by a recent exploratory study by Foddis et al. (2019) reporting an almost equal distribution among different PcomA phenotypes (no patent PcomA, unilateral patent PcomA or bilateral patent PcomAs). While the distribution of patent PcomA in C57BL/6 mice in that study differs from the report by McColl and colleagues and our own findings, it still confirms the general conclusion of a high degree of variability in C57BL/6 mice.

Randomization is a cornerstone of all trials of treatment effects. Randomization seeks to prevent bias in the allocation of subjects to treatment groups and is the prerequisite of any statistical testing. To ensure balanced distribution of all known and unknown covariates influencing the dependent variable(s), is the primary goal of randomization. Imbalance in covariates that strongly correlate and determine the outcome variable can lead to an overestimation of the treatment effect or to false negative results, depending on the direction of correlation and the level of imbalance. For clinical trials, methods to adjust for imbalances of known covariates both at the design stage (i.e., stratification) as well as at the stage of data analysis (i.e., by including the covariates in the statistical model) have been commonly adopted (Schneider et al., 2012). The risk of imbalance grows with increasing levels of variability of a given variable in the statistical population and with decreasing sample size. Smaller sample sizes are also associated with an increasing effect of any imbalance in covariates on precision, effect size and power (Button et al., 2013). In preclinical biomedical research, sample sizes are often under 10 animals per group (Button et al., 2013), increasing the impact of imbalance of covariates, especially those (such as PcomA patency) that are strongly correlated with the outcome parameter. We therefore argue that adjustments for imbalances in the distribution of animals with different PcomA statuses could be beneficial for all stroke studies using C57BL/6 mice.

In recent years, high-resolution mouse MRI has become widely available at almost all large stroke research centers. Beckmann and colleagues have demonstrated that high-resolution MRA can be used to non-invasively image cerebrovascular anatomy, including large collaterals in the circle of Willis in mice (Beckmann et al., 1999; Beckmann, 2000). Building on this work, we and others have refined MRA protocols to obtain high-resolution MRA in less than 10 min per animal, rendering the technique applicable for routine use in preclinical stroke research when MRI is included in the study design. Beckman and colleagues proposed using this method for selecting or stratifying animals according to the PcomA status. However, our current data on the inconsistent association of PcomA patency evaluated before fMCAo and the ischemic lesion size, as well as data reported in a recent study by Foddis and colleagues, highlight challenges of this approach (Foddis et al., 2019).

There are several possible explanations for the failure to predict lesion size from pre-fMCAo PcomA evaluation. It is possible that the resolution currently available in TOF MRA sequences is not sufficient to distinguish between smaller PcomA vessel diameters in preischemic conditions. This notion is support by our finding of a moderate, yet statistically not significant (*p* = 0.051), correlation of manual PcomA size ratings before fMCAo and infarct size 24 h after fMCAo, a signal not picked up by our semiquantitative score for PcomA patency. Studies using contrast-enhanced MRA with a cryogenically cooled MR coil and extended recording time demonstrated the technical possibility of imaging vessels with diameters of down to 40 μm (Klohs et al., 2012). Further studies using this technique would be needed to validate this hypothesis. Contrast-enhanced MRA, however, requires long sequences and is inherently invasive, rendering this method less suitable for routine use. In line with the findings reported in this study, Foddis and colleagues found PcomA to be small and not reliably identifiable on MRA in naïve conditions, while under postischemic conditions PcomA is recruited and reaches a vessel size of up to 60% of the basilar artery diameter (Foddis et al., 2019). Despite our finding of a moderate correlation of manual PcomA scores pre-fMCAo and semiquantitative PcomA scores after fMCAo, this might indicate that preischemic PcomA size is only one of several determinants of PcomA perfusion under ischemic conditions. Interindividual differences in collateral recruitment under hypoperfusion might also play a role. In fact, our finding of no reliable prediction of lesion size by preischemic PcomA scores suggests that not the preischemic anatomical size of the PcomA but the postischemic flow after collateral recruitment is what determines ischemic lesion size. Collateral status has recently been shown to be a key predictor of lesion size evolution in the first 24 h in humans (Rao et al., 2020). It can thus be hypothesized that postischemic PcomA flow as measured in this study is only a correlate of a general level of collateral recruitment in the individual animal determining the size of the ischemic lesion. In this scenario, interindividual differences concerning collateral recruitment might be more important for lesion development than the initial PcomA vessel size pre fMCAo. We therefore argue that using a measurement of the postischemic PcomA status by TOF MRA obtained 24 h after fMCAo as a covariate in the outcome analysis is most relevant to adjust for imbalances in interindividual differences of cerebrovascular anatomy and function.

The inaptitude to measure PcomA patency as a determinant of ischemic lesion size in naïve, pre-fMCAo condition is at the same time one to the strongest limitations of the proposed approach. In the case of a treatment paradigm with early treatment within the first 24 h or pre-fMCAo treatment paradigms, the interaction of the treatment and the PcomA size cannot be excluded. This is particularly problematic for treatments targeting collateral recruitment. In these cases, adjusting for PcomA patency bears the risk of reducing power to detect the treatment effect and cannot be recommended. For experimental paradigms with postischemic treatment randomization later than 24 h after fMCAo, the proposed approach would allow for combining stratified randomization for PcomA status and including PcomA status as a covariate using ANCOVA to control for imbalances of this important covariate. In our study, we focused on variability in acute lesion size as one of the most widely used outcome parameters in experimental ischemic stroke studies and did not include long-term behavioral outcomes. Impact of PcomA patency on long-term behavioral outcomes should be assessed in future studies to test robustness of the proposed approach. To further improve robustness of the described approach and reduce potential bias, we aim to eliminate the currently still required manual input in delineation of the target area and automatise area selection for future studies. A general limitation of the method used, is the indirect measurement of cerebral blood flow (CBF) by morphological TOF MRA imaging. To better assess functional patency and blood flow and relate it to morphological PcomA status, additional measurement of CBF by non-invasive aterial spin labeling (ASL) would be desirable. The scope of the presented work was to use highly standardized widely available sequences easily applicable to routine protocols. We thus did not implement ASL in the current study protocol.

In conclusion, we have shown that including a semiquantitative score of PcomA patency obtained 24 h after fMCAO in the statistical model can explain a large share of previously unexplained intersubject variability in lesion size and thus increase the power to detect treatment effects in preclinical stroke studies. The methods used are easy to implement for study designs with MR imaging. Based on the findings in this study, we therefore suggest to, (i) record an TOF MRA 24 h after fMCAo followed by semiquantitative PcomA measurement in any protocol evaluating experimental stroke therapies in the subacure phase after stroke, (ii) include the semiquantitative score of PcomA patency in the statistical model for outcome assessment. We believe that, mirroring standard procedures in clinical trials for which adjustment for known covariates has become state-of-the-art, similar practices should be adopted for preclinical trials to improve efficiency in therapy selection for clinical translation.

## Data Availability Statement

The datasets presented in this study can be found in online repositories. The names of the repository/repositories and accession number(s) can be found below: https://figshare.com/s/117e284677bdbedf08e7; https://figshare.com/s/d1a9072c420c43e d908e; and https://figshare.com/s/e8e03096082a1d5c0f4f.

## Ethics Statement

The animal study was reviewed and approved by the Landesamt für Gesundheit und Soziales Berlin.

## Author Contributions

SK, CA, UD, SM, CH, CJH, SPK, ME, and PB-S contributed to designing and planning the experiments as well as obtaining official licenses to conduct this study. SK performed the fMCAo surgeries and blinded outcome analyses. CA, SM, and CJH acquired the MRA and MRI datasets. SK, PB-S, and SPK contributed in image normalization and atlas registration. ME, CH, SK, and UD contributed in covariate analysis and simulation design. All authors contributed in drafting and revising the manuscript and approved the manuscript for publication.

## Conflict of Interest

The authors declare that the research was conducted in the absence of any commercial or financial relationships that could be construed as a potential conflict of interest.

## Footnotes

1 http://portal.brain-map.org/

2 Latest version available under https://github.com/ChariteExpMri/antx2

3 http://www.spmmouse.org/

4 http://elastix.isi.uu.nl/
